# Identification and Quantification of Enniatins and Beauvericin in Animal Feeds and Their Ingredients by LC-QTRAP/MS/MS

**DOI:** 10.3390/metabo9020033

**Published:** 2019-02-13

**Authors:** Josefa Tolosa, Yelko Rodríguez-Carrasco, Emilia Ferrer, Jordi Mañes

**Affiliations:** 1 Department of Food Chemistry and Toxicology, University of Valencia, Av/ Vicent A. Estellés, s/n, Burjassot, 46100 Valencia, Spain; emilia.ferrer@uv.es (E.F.); jordi.manes@uv.es (J.M.); 2 ProtoQSAR, CEEI, Avda. Benjamin Franklin 12, Desp. 8, Paterna, 46980 Valencia, Spain

**Keywords:** mycotoxins, feed, raw materials, enniatins, beauvericin, co-occurrence

## Abstract

Emerging fusariotoxins, mainly enniatins (ENNs) and beauvericin (BEA), are secondary toxic metabolites produced by *Fusarium* spp. and are widely distributed contaminants of cereals and by-products. Mycotoxin contamination in these products supposes an important risk to feed supply security in the feed industry due to the common use of cereals in feed formulations. Hence, continuous monitoring of both raw materials and feed mixtures is highly recommended as stated by sanitary authorities. Therefore, an analytical procedure based on liquid chromatography tandem mass spectrometry and an acetonitrile-based extraction followed by a d-SPE (QuEChERS) step for the simultaneous determination of emerging *Fusarium* mycotoxins was in-house validated and successfully applied to raw materials (*n = 39*) and feed manufactured with them (*n = 48*). The analytical method was validated following the European guidelines and satisfactory results were obtained. Both raw materials and complete feedstuffs showed mycotoxin contamination at incidences of 18% and 92%, respectively. ENN B was the most commonly found mycotoxin in the analyzed samples at concentrations up to several tens of µg/kg. On the other hand, the co-occurrence of mycotoxins was observed in 47% of samples, ENN B and BEA being the most common combination. These results highlight the necessity to take a vigilant attitude to monitor the occurrence of contaminants in raw materials and feedstuffs throughout the manufacturing chain and storage.

## 1. Introduction

Mycotoxins are toxic secondary metabolites of fungi belonging, essentially, to the *Aspergillus*, *Penicillium* and *Fusarium* genera. While *Aspergillus* and *Penicillium* are the main mycotoxigenic postharvest mold genera reported, *Fusarium* seems to be the most important preharvest contaminant in crops [1]. Fungi from *Fusarium* genera frequently colonize small-grain cereals and are associated with grain diseases, such as *Fusarium* head blight, as well as the accumulation of potentially toxic metabolites in the kernels [2]. These toxic metabolites can contaminate a wide range of commodities such as food crops including grains, fibers and other agricultural feedstock and raw materials. The most widespread *Fusarium* mycotoxins occurring in cereals and derivates at high levels are fumonisins (FBs), trichothecenes (TCs), zearalenone (ZEN) and the so-called emerging fusariotoxins such as enniatins (ENNs) and beuvericin (BEA) [1]. 

Despite many years of research and the introduction of good practices in the human and animal meal manufacture, mycotoxins continue to be a significant health concern in feed manufacture because cereals and cereal by-products are the main ingredients included in feed formulation. Among crops, corn and wheat are the most commonly used for this purpose, but also for human consumption; however, they are also used in other industrial procedures, such as ethanol and flour production, and the by-products obtained in these processes, mainly dried distillers’ grain with solubles (DDGS) are used to replace expensive grains in feed formulation [3]. 

Physical processing such as cleaning, sorting and milling results in a reduction of the concentrations of mycotoxins in the refined product, but the application of these procedures results in an increase of the mycotoxin amount in the cereal by-products. For this reason, although published data confirm that milling can reduce the mycotoxin concentration in-fraction intended for human consumption, the co-products obtained, mainly DDGS, concentrate the initial mycotoxin levels up to three times compared to original grain into fractions which are commonly used as animal feed [3,4,5]. This effect is also favored by the presence in feed formulation of fractions that contain the outer part of the grain, which contain a higher mycotoxin content due to the external fungal contamination [4,6]. This high mycotoxin content has been reported in diverse studies, which indicated that over 70% of the BEA and ENNs in the original grains were found in by-products after food processing [7,8]. As a result, the use of by-products of cereal processing from contaminated grains represents a potential risk to livestock. This may explain, in part at least, the higher proportions of poultry feed samples reported as being contaminated with BEA and ENNs compared to whole maize grain [9]. In this sense, the inclusion of cereals and their by-products, mainly DDGS, in animals’ diets must be carefully calculated since fungi can produce mycotoxins in all steps of both the food and feed chains [4,10,11]. Mycotoxin contamination in these by-products supposes an important risk to feed supply security in the feed industry due to the common use of cereals in feed formulations [1,3,5,12]. In fact, many studies have reported mycotoxin occurrence on different ingredients used in feed formulation and in finished feeds intended for terrestrial animals [13,14,15,16,17]. In summary, the inclusion of high amounts of vegetal origin sources, such as cereals and other raw materials, increases the risk of mycotoxin contamination [18] and, thus, the carry-over into edible tissues and/or animal byproducts, such as milk or eggs.

From a regulatory point of view, aflatoxin B_1_ (AFB1) is the only mycotoxin under the European Union feed regulation up to now (20 µg/kg in raw materials), as it has been classified as carcinogenic according to the International Agency for Research on Cancer (IARC) [19,20]. For other mycotoxins, mainly *Fusarium* mycotoxins, guidance values have been set for feed ingredients and finished feed, including deoxynivalenol (DON), zearalenone (ZEN), and the sum of fumonisin B_1_ and B_2_ (FB1 + FB2), whereas for the T-2 and HT-2 toxins, only indicative levels for cereal products have been set [21,22,23].

**Figure 1:**
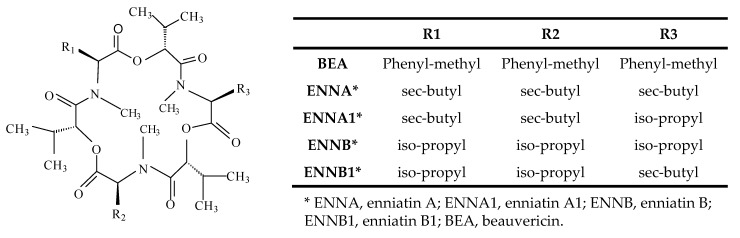
The structure of Enniatins (ENNs) and Beauvericin (BEA).

However, the limits for some emerging *Fusarium* mycotoxins have not been set, although their presence has been assessed by the European Food Safety Authority (EFSA) in both food and feeds in high levels (up to mg/kg or ppm) [24]. This is the case of ENNs and BEA. ENNs are structurally related mycotoxins representing a large group of cyclic hexadepsipeptides while BEA is a cyclic hexadepsipeptide belonging to the enniatin antibiotic family (Figure 1), which are primarily contaminants of cereals. BEA and ENNs are substantially stable during commercial cereal processing, including hot drying and ensiling procedures. Thus, they are widely present in livestock feeding as cereal grains or by-products resulting from cereal processing which are included in feedstuffs, and their presence in feed and raw materials has been reported in different studies [25,26,27]. In this sense, contents for enniatin B (ENN B) and BEA up to 2598 µg/kg and 988 µg/kg, respectively, have been reported [26]. Moreover, it should be taken into account that mycotoxin presence in livestock and poultry production may lead to economic losses and veterinary costs due to the negative effects on animal performance and the welfare of animals.

The European legislation on animal feed provides a legal framework for ensuring that feed does not suppose a risk to human or animal health. Nevertheless, the mycotoxin contents reported in the scientific literature indicate that sometimes the limits proposed for cereal-derived products by the European legislation may be not warranted. Therefore, the continuous monitoring and analysis of both raw materials and feed mixtures is highly recommended and, thus, included under specific projects [28], as, for example, in Spain, in the National Residue Monitoring Plan and the National Plan of Feed Inspection and Monitoring, focusing on the analysis performed on mycotoxins to which production animals are vulnerable.

From a toxicological point of view, it has been reported that BEA exerts cytotoxic activity against different animal and human cell lines, producing cellular damage by reactive oxygen species (ROS) generation and membrane lipid peroxidation (LPO), thus, producing oxidative stress. Moreover, the genotoxic activity of BEA has been also evidenced by different authors [29].

The ionophoric property seems to be the cause of BEA toxicity, as it also occurs in the case of ENNs. Due to the ionophoric properties, BEA is capable to promote the transport of mono- and divalent cations through membranes producing disturbances in the normal physiological cellular concentrations. 

Regarding ENNs, it has been demonstrated that they are capable of inducing cytotoxicity under *in vitro* conditions. They can disturb the normal cell proliferation due to their hydrophobic nature, which affects the ionic homeostasis by the formation of dimeric structures that transport monovalent ions across the cellular membranes and can be easily incorporated in biological membranes, mainly the mitochondrial membrane. The cell damage is produced mainly due to the ROS generation and the induction of LPO [30].

Therefore, the accumulation of mycotoxins in foods and feeds represents a major threat to human and animal health as they are responsible for many different toxicities including the induction of cancer, mutagenicity and estrogenic, gastrointestinal, urogenital, vascular, kidney and nervous disorders.

Based on the information mentioned above, the aim of the present study was to carry out a survey on ENN and BEA occurrence in different raw materials (*n* = 39) commonly used in animal diets and feed intended for different animal species (*n* = 48). To achieve this objective, a confirmatory method based on liquid chromatography-tandem mass spectrometry was in-house validated and then applied to 87 samples to further broaden the knowledge on emerging fusariotoxins occurrence and co-occurrence in feedstuffs and by-products.

## 2. Results and Discussion

### 2.1. Instrumental Optimization

The mycotoxin measurements were performed by an acetonitrile-based extraction followed by a d-SPE (QuEChERS) step followed by LC-MS/MS with a 3200 QTRAP® System AB Sciex (Applied Biosystems, Foster City, CA, USA) functioning as a triple quadrupole mass spectrometer detector (MS/MS). The mass spectrometric conditions were optimized by the direct infusion of individual working standard solutions, using an ESI source in both positive and negative modes. The results showed that the studied emerging fusariotoxins have higher peaks and response values in the positive ionization mode ([M + NH_4_]^+^). The most intense precursor ions were selected and the cone voltage was optimized for each target mycotoxin, with the mass spectrometer operating in the product ion scan mode. Subsequently, collision energies were optimized for each transition and the product ions were selected for mycotoxin quantification and qualification. An entrance potential (EP) of 10 V and a collision cell exit potential (CXP) of 4 V were set for all the analytes. The final selection of the selected reaction monitoring (SRM) transitions in positive ion mode for each studied compound and the optimal MS parameters, namely, declustering potential (DP), collision energy (CE) and cell exit potential (CXP) are shown in Table 1. 

**Figure 2:**
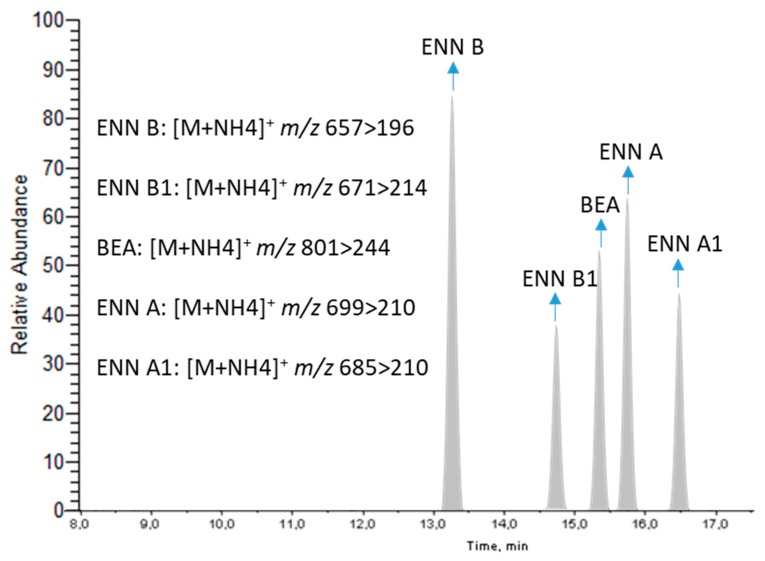
The LC-MS/MS Chromatogram for Target Emerging Fusariotoxins at a Concentration of 10xLOQ.

Previous studies have shown that adding a buffer and/or volatile acid into the mobile phase is beneficial to improve the efficiency of compound ionization. In this work, chromatographic behaviors of target fusariotoxins were comparatively investigated in the elution solution of methanol/water with and without ammonium formate (5 mM) and formic acid (0.1%). The results showed an improvement in both the peak shape and the analyte response by adding ammonium formate and formic acid. Therefore, methanol/water containing ammonium formate (5 mM) and formic acid (0.1%) were selected as the mobile phase in this study. Figure 2 shows the LC-MS/MS chromatograms for target emerging fusariotoxins in the ESI positive mode.

### 2.2. Method Validation

The specificity and selectivity of the method rely on the chromatographic retention time of each analyte and on the SRM transition used. The peaks for the studied analytes in the samples were confirmed by comparing the retention time of the peak with those of standards at the maximum tolerance of ±0.2 min or ±50% of the peak width at half height, recognizing both the quantitation (Q) and confirmation (q) transitions, and matching the ion ratio. In addition, no interference peaks were observed at retention times of the target compounds in blank samples. Concerning linearity, all the studied mycotoxins showed correlation coefficients (R^2^) greater than 0.990 over the working range (0.1–200 µg/kg). Co-eluting matrix components can negatively influence the accuracy of quantitative methods through signal ion suppression or enhancement (SSE) in the ion source; thus, the effects of the possible matrix mismatch were assessed. The results showed a significant signal suppression for the studied analytes (from 13 to 29%) and therefore matrix-matched calibration curves were used for a quantitative purpose. The sensitivity of the method was assessed by the limit of detection (LOD) and limit of quantitation (LOQ). LODs and LOQ_S_ were in a range from 0.2 to 1.0 μg/kg, and from 1.0 to 5.0 μg/kg, respectively. The trueness of the method, expressed as a recovery of analytes, was evaluated at three spiking levels (low level: LOQ; intermediate level: 10xLOQ; high level. 100xLOQ) and the results showed a recovery range from 86 to 98%, from 112 to 136%, and from 89 to 117%, at low, intermediate and high fortification levels, respectively (Table 2). As far as precision is concerned, the repeatability studies showed a relative standard deviation (RSD) lower than 5% at two spiking levels, whereas RSDs lower than 15% were obtained in the reproducibility studies. 

Based on the before mentioned validation results, the proposed procedure is suitable for its purpose since it is a specific, sensitive, accurate, precise and robust method. The key performance characteristics fulfill the criteria set at the Commission Decision 2002/657/EC [31] and guidance document on the identification of mycotoxins in food and feed SANTE/12089 /2016 [32]. 

### 2.3. The Natural Occurrence of Mycotoxins in Raw Materials

The natural occurrence of ENNs and BEA was investigated in raw materials (*n = 39*) commonly used in feed manufacturing. The results revealed that the most prevalent mycotoxins were ENN B and BEA (18%), followed by ENN B1 (6%), while ENN A and ENN A1 were not detected in any of the analyzed samples. These results are in agreement with data reported in previous studies, ENNs type B being the major occurring mycotoxin in cereals and by-products in the following decreasing order: ENN B > ENN B1 > ENN A1 > ENN A. For instance, Mortensen et al. [33] reported ENN B contamination in all DDGS samples (*n = 7*) in by-products for animal feed collected from the official control in Denmark during 1998–2009. Similarly, Uhlig et al. [2], reported ENN B contamination in all analyzed samples of barley (*n = 75*), wheat (*n = 80*) and oat (*n = 73*) from Norway. This trend was also reported by Van Pamel et al. [1] who reported ENNs and BEA as the most frequently detected mycotoxins in all maize silage samples *(n = 10*) from Belgium, at trace levels (under the LOQ), with LOQ values of BEA 44 µg/kg, 48 µg/kg for ENN A, 43 µg/kg for ENN A1, 52 µg/kg for ENN B and 47 µg/kg for ENN B1. Those results were similar to those reported by Sørensen et al. [25] who found that up to 90% of the contaminated samples by ENN B in 2005 and in 100% in 2006 (*n = 30* and *n = 43* in 2005 and 2006, respectively), in maize silage from Denmark. On the other hand, ENNs type A (ENN A and ENN A1) were not detected in any sample of raw materials included in this study. In accordance with these results, Sørensen et al. [25], did not detect ENN A and ENN A1 in grain samples analyzed nor in 3-month-old silage stacks from the whole maize. Nonetheless, Streit et al. [17] reported ENN A1 contamination in 95% of raw materials analyzed (*n = 48*) and ENN A in 87% of samples. In those feed and raw materials, the occurrence of ENN type B (95%) and BEA (98%) was also reported.

Regarding the concentration of the emerging fusariotoxins found in raw materials here analyzed, ENN B was found in a range between 1.3 and 75.6 µg/kg, whereas ENN B1 contents ranged between 36.3 and 113.2 µg/kg, and BEA was found from 3.0 to 64.8 µg/kg. These results are in the same range than that reported by Shimshoni et al. [34] in corn and wheat silage from Israel, with mean values of 0.3 µg/kg, 0.9 µg/kg and 66 µg/kg, respectively, in corn and 0.3 µg/kg, 0.9 µg/kg and 5 µg/kg, respectively, in wheat samples. In addition, the results obtained in our study were in accordance with that reported by Warth et al. [26] in grain-based processed foods containing wheat. These authors found maximum contents of 16.4 µg/kg, 21.4 µg/kg and 47 µg/kg for ENN B, ENN B1 and BEA, respectively, in samples from Burkina Faso and 0.9 µg/kg, 4.1 µg/kg and 486 µg/kg, respectively, in samples collected in Mozambique. In spite of that, the above-reported concentrations significantly differ (*p* < 0.05) from those obtained in other studies performed in northern European regions. In these colder regions, higher contents have been reported compared to warmer zones. In this sense, Jestoi et al. [35] reported the presence of ENN B and ENN B1 in 100% of the analyzed raw material (mainly wheat and barley) from Finland (*n = 38*), reaching maximum levels up to 3980 µg/kg and 3240 µg/kg, respectively, in barley samples analyzed. These high levels have been also observed by Uhlig et al. [2], who found a maximum concentration of 5800 µg/kg for ENN B in wheat samples from Norway and by Sørensen et al. [25] who reported up to 2600 µg/kg of ENN B in whole fresh maize from Denmark. Similar results were also reported by Habler and Rychlik [36] in cereals from Germany; and Zachariasova et al. [27] reported average concentrations of ENN A and ENN B at 615 µg/kg in hay samples (*n = 4*) and 748 µg/kg in wheat-based DDGS samples (*n = 16*) from the Czech Republic, respectively. This fact could be explained due to the climatic conditions favorable to the proliferation of *Fusarium* spp. which produces ENNs. For BEA, the highest contents were found in rice bran and corn pulp samples, with an incidence of 25% and 50%, respectively (Table 3). According to Streit et al. [17], BEA was found in 98% of feed and raw material samples analyzed with a maximum content of 2326 µg/kg. In the survey reported by Lee et al. in Korea [37], 27% of feed ingredients were contaminated with BEA, at an average concentration of 480 µg/kg. 

### 2.4. Natural Occurrence of Mycotoxins in Feed

The results of acquired feedstuffs (*n = 48*) showed that 92% of the samples were contaminated with emerging fusariotoxins, ENN being B the most commonly found mycotoxin (89%), followed by ENN B1, BEA and ENN A1 (64%, 62% and 41.5%, respectively). ENN A was not detected in any of the analyzed samples. The results obtained are shown in Table 4. The highest average contents were found for ENNB in feed intended for rabbits, sheep, beef, dairy cattle and swine, which could be explained because of the contamination levels of the raw materials (pelleted diet and dried forages, mainly from cereals and vegetal protein). These results suggest that raw materials included in feed manufacture intended for those species showed higher contamination or were included in a higher proportion than feed intended for other species [38]. Nonetheless, there were no statistical differences (*p* > 0.05) among the contamination levels of the different feedstuffs.

The concentration range for the detected mycotoxins was the following: ENN A1 from 8.1 to 13.1 µg/kg; ENN B from 2.0 to 89.5 µg/kg; ENN B1 from 7.4 to 28.8 µg/kg; and for BEA, the concentration range was comprised between 4.6 and 129.6 µg/kg. The concentrations found in this study were in accordance with those reported by Warth et al. [26] in feed from Mozambique (*n = 10*), where ENN B and ENN B1 showed concentrations in a range between 2.2 and 114.0 µg/kg and 0.1 to 94.4 µg/kg, respectively; whereas BEA was detected in 100% of the analyzed samples at concentrations ranging from 3.3 to 418.4 µg/kg. In that study, the lowest contents corresponded to ENN A (from 0.6 to 7.9 µg/kg) and ENN A1 (from 3.4 to 43.9 µg/kg). 

In the analyzed samples here, ENNs and BEA contamination were found in both raw materials and feedstuffs being higher in the latter. It could be justified taken into consideration that compound feed is particularly vulnerable to mycotoxin contamination as it typically contains a mixture of several raw materials, mainly cereals and seed proteins. Even raw materials are subjected to different processes, mainly pelletization and/or extrusion, which are supposed to reduce the initial mycotoxin concentration. Inappropriate conditions during feed storage or manufacture can result in fungal contamination and, consequently, mycotoxin production [9,11,26,38,39,40].

Regarding the different species that the feed is intended for, lower contents were reported for cat feedstuffs. This fact is probably due to the analysis of “grain free” feeds for cats. The main ingredients used in the elaboration are fish and fish by-products (herring, sole, hake), among other vegetal ingredients such as legumes or fruits.

Bovine feed samples (*n* = 8) were intended for different stages of production, mainly fattening calves (*n* = 4) and dairy cattle (*n* = 4). The results showed that two out four samples of feed intended to fattening calves presented higher contents for the analyzed mycotoxins compared with the other two samples. The highest incidence was for ENN B (88%), followed by ENN B1 and BEA (75%) and finally ENN A1 (63%). Regarding ENN and BEA contents, it was observed that the analyzed feedstuffs for dairy cattle showed higher BEA contents (51.4 µg/kg) compared to those obtained in fattening cattle feed. These results are in agreement with those presented by Lee and collaborators [37], in which a higher mean BEA content was observed in feed intended for dairy cattle (720 μg/kg) than in beef cattle (430 μg/kg), although the contents were much higher than those reported in the present study. In the study carried out by Zachariasova et al. in the Czech Republic [27], the maximum contents of 236 μg/kg of ENN B and 34 μg/kg of BEA were described in feed for dairy cows.

Regarding the contents obtained in the samples of feed destined to the ovine livestock (*n* = 13), samples included in the study were intended for lambs (*n* = 11) and dairy sheep (*n* = 2). The results showed that the highest incidence of contamination corresponded to ENN B (86%), followed by BEA (79%), ENN B1 (72%) and ENN A1 (57%). The contents obtained were similar for ENNs, however, lower BEA contents were reported in dairy feedstuffs (8.1 μg/kg).

In the porcine section, analyzed feedstuffs were intended for different stages of production, mainly breeding, farrowing and fattening. As it can be observed in Table 4, the highest incidence and the highest contents corresponded to the ENN B. In feed intended for adult pigs and breeder sows, no ENN A1 contents were detected, whereas, in feed intended for piglets and fattening pigs, BEA was not detected. However, according to Lee and collaborators [37], in a study carried out in Korea, samples of feed destined for pigs showed average levels of 740 μg/kg of BEA. However, the data obtained by these authors indicate higher levels in feed destined for piglets, in contrast to the present study, in which the feed contents destined for fattening pigs were higher. These results are in agreement with those described by Zachariasova et al. [27] in a study conducted in the Czech Republic, in which the ENN B reached maximum levels of up to 799 μg/kg in swine feed samples.

It has to be highlighted that a significant co-occurrence of BEA and ENNs (47% of samples) was found in the present survey. The presence of other *Fusarium* mycotoxins in raw materials has been reported by different authors, mainly FBs, TCs and ZEN [41,42,43], but the results obtained here also highlight that the emerging *Fusarium* mycotoxins can be found in feedstuff commodities simultaneously. Therefore, special attention should be paid to the co-occurrence of *Fusarium* mycotoxins since additive and/or synergistic effects could occur, as recently observed in in vitro studies by Prosperini et al. [44].

## 3. Materials and Methods 

### 3.1. Sampling

A total of 87 samples were analyzed for mycotoxin determination in this work. Samples were classified as follows: 48 feedstuffs and 39 raw materials commonly used as ingredients in feed manufacture. All of them were purchased from farms and feed factories located in the Valencia province, Spain. 

Feed intended for different animal species was used as the criteria for samples sub-classification. Therefore, feedstuffs were grouped as follows: intended for ovine (*n = 13*); intended for poultry (*n = 11*); intended for bovine (*n = 8*); intended for domestic animals such as dogs and cats (*n = 6*); intended for swine (*n = 4*); intended for horses (*n = 3*); intended for rabbits (*n = 2*); and intended for caprine (*n = 1*). The raw materials analyzed were: barley (*n = 10*), meals (*n = 7*, mainly sunflower, rapeseed and soybean), gluten feed (*n = 5*, mainly wheat and corn gluten), rice bran (n = 4), corn pulp (*n = 4*), wheat (*n = 3*), maize *(n = 2*), alfalfa *(n = 3*) and sugar beet pulp (*n = 1*). These raw materials were selected by taking into account those with a high inclusion percentage in feed elaboration. The percentage of inclusion depended on the species the feed is intended for and the feed formulation. 

All samples were homogenized in a food blender and then kept in dark and dry conditions at 4 °C until analysis.

Raw materials and feeds manufactured with them have been collected in factories from the region where the study has been performed. In this sense, feed production is intended for common livestock of the region. This is the reason why there are scarce samples intended for some animal species (caprine) compared to others, such as ovine and poultry.

### 3.2. Chemicals and Reagents

All solvents (acetonitrile (MeCN) and methanol (MeOH)) were acquired at Merck (Darmstadt, Germany). Deionized water (<18 MΩ cm resistivity) was obtained from a Milli-Q water purification system (Millipore Corporation, Bedford, MA, USA). Formic acid (HCOOH) and ammonium formate (HCOONH_4_, 97%) were supplied by Sigma-Aldrich (Madrid, Spain). All solvents were filtered through a cellulose filter of 0.22 µm (Membrane Solutions, Plano, TX, USA) before use. The stock standards of ENNs and BEA were purchased from Sigma-Aldrich (St. Louis, Missouri, USA). Anhydrous magnesium sulfate was obtained from Alfa Aesar GmbH & Co. (Karlsruhe, Germany); sodium chloride was purchased from Merck and C18 was purchased from Phenomenex (Torrance, CA, USA). 

### 3.3. Preparation of Standard Solution and Spiking of Blank Samples

Individual stock solutions of BEA and ENNs with a concentration of 1000 µg/mL were prepared in MeCN. They were stored in darkness conditions in glass-stoppered bottles at −20 °C. The working standard solutions consisting of individual compounds were prepared by the appropriate dilution of the stock solutions for spiking procedures and calibration curves. Samples of commercial feed and their ingredients containing none of the studied mycotoxins were used as a blank matrix for spiking experiments as well as for quality control. The spiked samples were left for overnight equilibration.

### 3.4. Sample Preparation

Two grams of the homogenized matrix were weighed into a 50 mL polypropylene (PP) tube and 10 mL of water containing 2% formic acid and 10 mL of MeCN were added and vigorously shaken for 30 min on a horizontal shaker (IKA, Staufen, Germany). Then, 1 g NaCl and 4 g of MgSO_4_ were added and the tube was vortexed for 30 s and then centrifuged for 5 min at 2336 g and 4 ˚C (Eppendorf, Germany). Two mL of MeCN extract was collected and submitted to a dispersive solid phase extraction (dSPE, 15-mL PP tube), employing 0.1 g of C18 silica sorbent and 0.3 g of MgSO_4_ and then centrifuged for 5 min at 1413 g and 4 ˚C. Finally, the purified extract was filtered through a 0.22 µm nylon filter and transferred into a vial for LC-MS/MS analysis.

### 3.5. LC-MS/MS Equipment and Conditions

The instrumental analysis was achieved on liquid chromatography coupled with a tandem mass spectrometry (LC-QTRAP/MS/MS) system. Chromatographic separation of the analytes was conducted at 25 ˚C using an Agilent 1200 chromatographic system (Agilent Technologies, Palo Alto, CA, USA) with a binary pump and automatic injector. A reverse-phase Gemini-NX C18 (150 mm × 2 mm I.D., 3 μm particle size) analytical column (Phenomenex, Barcelona, Spain) was used. The analytical separation was performed using a gradient elution of 95% of phase A (water) and 5% of phase B (MeOH), both with 5 mM of ammonium formate and 0.1% formic acid, increasing linearly to 95% B for 10 min; then, decreasing linearly to 80% B for 5 min, and then gradually up to 70% B for 6 min. Finally, for the last 3 min, the initial conditions were maintained. The flow rate was maintained at 0.2 mL/min.

For the mass spectrometry analysis, a 3200 QTRAP® mass spectrometer operated in the Selected Reaction Monitoring (SRM) mode (AB Sciex, Foster City, CA, USA) equipped with a turbo electrospray ionization (ESI) interface was used. The numerous heteroatoms in BEA and ENNs are the main reason why they ionize very well in the positive electrospray mode, and for this reason, the analysis was performed in positive ion mode (ESI+) and the ESI source values were as follows: capillary voltage: 3.50 kV; source temperature: 120 ˚C; desolvation temperature: 400 ˚C; cone gas: 50 L/h; desolvation gas (nitrogen 99.99% purity) flow: 800 L/h. The QTRAP® analyzer combines a fully functional triple quadrupole and a linear ion trap mass spectrometer within the same instrument. The resolution for the first and third quadrupoles was set to 12.0 (unit resolution); the ion energy to 0.5; the entrance and exit energies to 5 and 3, respectively; the multiplier to 650; the collision gas (argon 99.99% purity) pressure to 3.83 ×10^−3^ mbar; the interchanel delay to 0.02 s; the total scan time to 1.0 s; and the dwell time to 0.1 ms. SRM optimized parameters were calculated in triplicate (cone voltages, collision energies and precursor and product-ions selected) and are shown in Table 1.

### 3.6. Method Validation and Quality Assurance/Quality Control (QA/QC)

The method performance parameters were determined according to European guidelines [28]. The method was validated for mycotoxin standards with regards to its selectivity, specificity, linearity, matrix effect, sensitivity, trueness and precision. 

Feed and raw material samples in which mycotoxins were not detected, were mixed to obtain an individual composite sample. This composite was used as the blank matrix to carry out both the matrix-matched calibration curves and the spiked samples used for recovery and quality control assays. The selectivity and specificity of the method were ascertained by analyzing the standard solutions and the spiked samples. The peaks for the studied compounds in the samples were confirmed by comparing the retention time of the peak with those of the standard solution, as well as by recognizing both the precursor and product ions and their ratio. The linearity and matrix effects were studied using standard solutions in a neat solvent and matrix-matched calibrations. The calibration curves in both the pure solvent and matrix were constructed by plotting the signal intensity against analyte concentrations at eight levels (from 0.1 µg/kg to 200 µg/kg). The calibration curves were prepared in triplicate. To assess the matrix effects, the ratios between the slope of matrix-matched (A) and the slope of external calibration (B) were obtained. Thus, the ratio (A/B) × 100) is defined as the matrix effect and expressed as the signal suppression/enhancement (SSE, %). SSE values < 100% indicate signal suppression; >100% signal enhancement; whereas values equal to 100% indicate no matrix effect. Sensitivity was evaluated by the limits of detection (LOD) and the limits of quantitation (LOQ). LOD and LOQ were calculated as the lowest addition level constructed in the matrix-matched extract, corresponding to a signal to noise ratio of at least 3:1 and 10:1, respectively. The trueness and precision studies were evaluated by spiking the standard solution to blank samples at two concentration levels (10 x LOQ and 100 × LOQ). Trueness was expressed as a percentage of recovery. Precision was verified by three determinations on the same day (repeatability) and on three non-consecutive days (reproducibility). 

Analytical quality control samples were evaluated during the method validation and analysis of samples according to the guidance document on the identification of mycotoxins in food and feed SANTE/12089 /2016 [32]. 

### 3.7. Statistics and Data Analysis

All experiments were performed in triplicate, and the results were expressed as the average values ± relative standard deviation (RSD, %). A Student’s t-test statistical analysis was performed for data evaluation; *p* values < 0.05 were considered significant. Tests were carried out by using IBM SPSS 24.0 (SPSS Inc., Chicago, IL, USA).

## 4. Conclusions

In this work, an LC-MS/MS method for the simultaneous determination of emerging *Fusarium* mycotoxins, namely, enniatins and beauvericin, in raw materials and complete feedstuffs was in-house validated. The results showed that the proposed analytical procedure was accurate (recovery range from 89 to 136% for the vast majority of analytes) and precise (RSDs < 15%, and sensitive (LODs from 0.2 to 1.0 μg/kg) to fulfill the criteria established in European guidelines. The validated method was successfully applied to 87 samples of raw materials and feedstuffs to monitor the occurrence levels of the studied mycotoxins. The results showed that ENNs and BEA were present in both raw materials and feeds, ENN B detected in up to 92% of samples. Mycotoxin concentrations found in analyzed samples varied depending on the type of sample, being those with a high level of cereal inclusion the most contaminated. In addition, the co-occurrence of mycotoxins was frequently detected in samples (47%). These data indicate that the contamination of feedstuffs with only one mycotoxin is rare and that mycotoxins occur more frequently together, representing a risk for animals. Thus, to monitor the occurrence of mycotoxins in raw materials and feedstuffs throughout the manufacturing chain and storage, we need to guarantee the safety of animals and trade requirements.

## Figures and Tables

**Table 1:** The optimized MS/MS Parameters.

Mycotoxin	RT (min)	Precursor Ion	Product Ions	DP^c^ (V)	CE^d^ (V)	CXP^e^ (V)
ENN A	15.8	699.400	228q^a^/210Q^b^	76/76	59/35	16/14
ENN A1	16.5	685.400	214q^a^/210Q^b^	66/66	59/37	10/8
ENN B	13.3	657.300	214q^a^/196Q^b^	51/51	59/39	10/8
ENN B1	14.7	671.200	228q^a^/214Q^b^	66/66	57/61	12/10
BEA	15.3	801.200	784Q^a^ /244q^b^	116/116	39/27	6/10

^a^ q, confirmation; ^b^ Q, quantitation; ^c^ DP, declustering potential; ^d^ CE, collision energy; ^e^ CXP, cell exit potential.

**Table 2:** The method performance.

Parameters	Recovery, % (RSD_R_, %; *n* = 9)			LOD	LOQ
	LOQ (ng/g)	10xLOQ (ng/g)	100xLOQ (ng/g)	(ng/g)	(ng/g)
ENN A	95 (12)	112 (10)	93 (8)	1.0	5.0
ENN A1	91 (8)	95 (9)	89 (6)	0.2	1.0
ENN B	89 (9)	98 (8)	97 (5)	0.2	1.0
ENN B1	98 (10)	105 (9)	94 (7)	0.2	1.0
BEA	86 (7)	136 (15)	117 (12)	1.0	5.0

RSD_R_: relative standard deviation (reproducibility).

**Table 3:** The Enniatin and Beauvericin contents in the raw materials analyzed (*n* = 39). The results are expressed as an average (Concentration Range) in µg/kg.

Raw Material (Number of Samples)	ENN A	ENN A1	ENN B	ENN B1	BEA
Wheat (*n* = 3)	*nd*	*nd*	50.2 (50.2)	36.3 (36.3)	*nd*
Maize (*n* = 2)	*nd*	*nd*	*nd*	*nd*	*nd*
Alfalfa (*n* = 3)	*nd*	*nd*	75.6 (75.6)	113.2 (113.2)	6.0 (6.0)
Sugar beet pulp (*n* = 1)	*nd*	*nd*	*nd*	*nd*	3.0 (3.0)
Barley (*n* = 10)	*nd*	*nd*	1.3 (1.3)	*nd*	*nd*
Rice bran (*n* = 4)	*nd*	*nd*	*nd*	*nd*	64.8 (64.8)
Corn pulp (*n* = 4)	*nd*	*nd*	1.8 (1.3–2.2)	*nd*	29 (20.4–37.8)
Meals (*n* = 7)	*nd*	*nd*	*nd*	*nd*	*nd*
Gluten feed (*n* = 5)	*nd*	*nd*	*nd*	*nd*	*nd*

nd: not detected.

**Table 4:** The Enniatin and Beauvericin Contents in the feed samples analyzed (*n* = 48). The results are expressed as an average (concentration range) in µg/kg.

Animal Specie (Number of Samples)	ENN A	ENN A1	ENN B	ENN B1	BEA
Bovine (*n* = 8)	*nd*	9.7 (8.5–10.7)	24.1 (2.4–41.6)	15.2 (10.8–20.2)	27.4 (20.7–51.4)
Ovine (*n* = 13)	*nd*	10.2 (8.1–13.1)	32.4 (2.0–89.5)	16.7 (9.4–28.8)	32.6 (8.1–129.6)
Caprine (*n* = 1)	*nd*	8.4 (8.2–8.5)	16.8 (8.3–23.9)	12.7 (10.8–15.0)	13.9 (4.6–23.2)
Horses (*n* = 3)	*nd*	9.4 (8.7–10.1)	21.8 (6.0–43.8)	13.6 (10.0–15.5)	19.0 (8.2–29.8)
Porcine (*n* = 4)	*nd*	10.5 (9.1–11.9)	32.2 (22.1–55.1)	17.0 (14.1–24.0)	10.2 (5.7–14.6)
Poultry (*n* = 11)	*nd*	9.7 (8.1–11.9)	18.4 (3.0–51.1)	15.3 (7.4–23.1)	15.8 (8.1–23.8)
Rabbits (*n* = 2)	*nd*	11.8 (11.8)	47.4 (44.5–50.3)	23.5 (23.3–23.6)	13.5 (13.5)
Dogs (*n* = 3)	*nd*	*nd*	15.4 (7.5–24.8)	10.1 (10.1)	30.9 (21.3–40.5)
Cats (*n* = 3)	*nd*	*nd*	6.7 (6.7)	8.9 (8.9)	*nd*

nd: not detected.
